# MRI Markers of Small Vessel Disease and the APOE Allele in Cognitive Impairment

**DOI:** 10.3389/fnagi.2022.897674

**Published:** 2022-07-13

**Authors:** Mana Shams, Sara Shams, Juha Martola, Lena Cavallin, Tobias Granberg, Magnus Kaijser, Max Wintermark, Eric Westman, Peter Aspelin, Maria Kristoffersen Wiberg, Lars-Olof Wahlund

**Affiliations:** ^1^Department of Clinical Neuroscience, Karolinska Institutet, Stockholm, Sweden; ^2^Department of Radiology, Karolinska University Hospital, Stockholm, Sweden; ^3^Division of Neuroradiology, Department of Radiology, Stanford University Hospital, Stanford, CA, United States; ^4^Department of Neurobiology, Care Sciences and Society, Karolinska Institutet, Stockholm, Sweden; ^5^Division of Clinical Geriatrics, Karolinska University Hospital, Stockholm, Sweden

**Keywords:** cerebral small vessel disease, cerebral amyloid angiopathy, hypertensive vasculopathy, apolipoprotein E, dementia, Alzheimer's disease, magnetic resonance imaging

## Abstract

**Objective:**

The apolipoprotein E *(APOE)* ε4 allele is the main genetic risk factor for dementia and Alzheimer's disease (AD), but the underlying mechanism for the increased risk is not well understood. Cerebral small vessel disease (SVD) is prevalent among patients with cognitive impairment and is thought to play an important role in the pathophysiology of dementia. We aimed to investigate the association between the *APOE* ε genotype and magnetic resonance imaging (MRI) markers of SVD in a memory clinic population.

**Material and Methods:**

This is a cross-sectional study with a total of 520 patients undergoing dementia investigation, including an MRI brain scan and *APOE* genotyping in all patients enrolled, and cerebrospinal fluid (CSF) analysis for routine AD biomarkers in 399 patients. MR images were assessed for markers of SVD: cerebral microbleeds (CMBs), cortical superficial siderosis, intracerebral hemorrhage, white matter hyperintensities, lacunar infarcts, and enlarged perivascular spaces.

**Results:**

Apolipoprotein E carriers with AD had a higher number of CMBs when looking at all brain regions and lobar brain regions (*p* < 0.001). A lower number of CMBs were seen in *APOE* ε2 (*p* < 0.05), ε3 and ε3/3 carriers (*p* < 0.001) when looking at all brain regions. A higher number of CMBs in deep and infratentorial regions were seen in *APOE* ε2 and ε3 (*p* < 0.05). In *APOE* ε4/4 carriers, CMBs, cortical superficial siderosis, white matter hyperintensities, and enlarged perivascular spaces were associated with lower levels of CSF amyloid β (Aβ) 42 in the whole cohort, and in individuals with AD and mild cognitive impairment (*p* < 0.05).

**Conclusion:**

Apolipoprotein E ε4 is associated with MRI markers of SVD related to amyloid pathology, specifically CMBs and Aβ42 plaque formation in the brain, as reflected by decreased CSF Aβ42 levels, whereas *APOE* ε3 and ε2 are associated with the markers of hypertensive arteriopathy, as reflected by the association with CMBs in deep and infratentorial brain regions.

## Introduction

Cerebral microbleeds (CMBs), cortical superficial siderosis, white matter hyperintensities, enlarged perivascular spaces, and lacunar infarcts are all seen as markers of small vessel disease (SVD) on magnetic resonance imaging (MRI) (Feldman et al., 2008; Doubal et al., 2010; Braun and Schreiber, 2013; Charidimou et al., 2013). CMBs have been specifically hypothesized to play an important role in the Alzheimer's pathophysiology (Cordonnier and van der Flier, 2011), are common in a memory clinic population (Cordonnier et al., 2006), and can be seen as a hypointense dots on hemosiderin sensitive MRI sequences and histopathologically as foci of hemosiderin deposits in the brain parenchyma (Werring et al., 2010). The main causes of CMBs are hypertensive arteriopathy, causing deep and infratentorial CMBs, and cerebral amyloid angiopathy, causing lobar CMBs (Werring, 2011). The topography of CMBs may thus help in understanding the underlying pathology. Cortical superficial siderosis is a subpial deposition of hemosiderin and has been linked to cerebral amyloid angiopathy (Feldman et al., 2008). White matter hyperintensities are seen as hyperintensities on T2-weighted and fluid attenuated inversion recovery (FLAIR) MRI sequences and are thought to be an expression of chronic white matter hypoperfusion (Brun and Englund, 1986; Pantoni, 2010). White matter hyperintensities have shown to predict the rate of cognitive decline in patients with Alzheimer's disease (AD) (Brickman et al., 2008). Enlarged perivascular spaces (EPVS) are perivascular cavities, which are fluid filled invaginations of the subarachnoid space, surrounding the penetrating vessels as they pass along and penetrate the subarachnoid space through the brain parenchyma (Braffman et al., 1988; Doubal et al., 2010).

All the abovementioned markers are demonstrated in patients with dementia (Werring, 2011; Zonneveld et al., 2014). MRI markers of SVD have shown to be especially prevalent in patients with vascular dementia and AD, and in AD, almost all patients are thought to suffer from cerebral amyloid angiopathy (Jellinger, 2002; Smith and Greenberg, 2009; Shams et al., 2014). However, although SVD MRI markers have been suggested to play an important role in dementia, their respective roles in dementia pathophysiology still remain unclear (Roher et al., 2003; Cordonnier and van der Flier, 2011).

The apolipoprotein E (*APOE)* allele is of importance in the development of sporadic and late AD (Verghese et al., 2011). The risk of AD in *APOE* allele carriers has been reported in the following order: ε4>ε3>ε2. Heterozygous or homozygous carriers for the *APOE* ε4 allele have an increased risk for late onset AD by 3- or 12-fold, respectively (Verghese et al., 2011). In the world of SVD, the ε4 allele is a risk factor for cerebral amyloid angiopathy and predisposes to intracerebral hemorrhage (Greenberg et al., 1995; Verghese et al., 2011; Schilling et al., 2013). Nevertheless, the role of the *APOE* ε4 allele in SVD and dementia is still not well understood.

We aimed to increase the understanding of SVD and *APOE* genotype in dementia by studying a large memory clinic population, focusing on groups with a clinical continuum of increasing AD pathology, from subjective cognitive impairment (SCI) to mild cognitive impairment (MCI) and AD. We hypothesized that ε4 carriers, in contrast to ε3 and ε2 carriers, would have accentuated markers of SVD related with cerebral amyloid angiopathy, especially patients with AD.

## Materials and Methods

### Study Population

**Figure 1:**
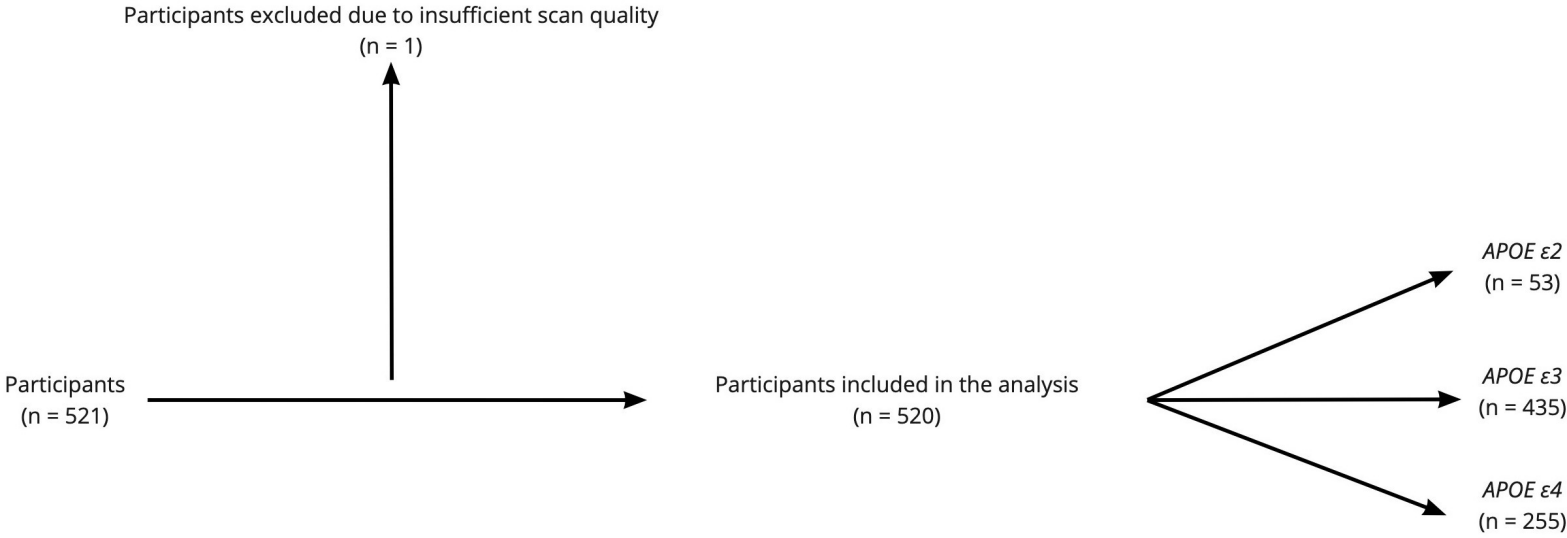
Flow diagram of the participants enrolled in the study. Among the 520 participants included in the analysis, 399 participants had cerebrospinal fluid (CSF) analysis for routine Alzheimer's disease (AD) biomarkers.

This study is part of the Karolinska Imaging Dementia Study (KIDS), a memory clinic based cross-sectional study on SVD in cognitive impairment. In total, 521 consecutive patients were enrolled, and all patients had been undergoing dementia investigation with accompanying *APOE* allele analysis and MRI scans at the memory clinic and radiology department, Karolinska University Hospital, between 01/01/2006 and 01/01/2012. Exclusion criteria for all patients were insufficient scan quality on the MRI and a history of traumatic brain injury. In our study, one patient was excluded due to poor scan quality on MRI, leading to a final cohort of 520 patients with 5 different diagnostic categories. The diagnostic category, “Other Disorders” (*n* = 38), was, however, discarded due to the heterogeneous nature of this group of patients, which limited statistical analysis. The diagnosis was set based on the ICD-10 criteria by an experienced memory clinic team consisting of geriatricians, neuropsychologists, neurophysiologists, and neuroradiologists after the entire clinical picture had been considered. The ICD-10 code used for MCI was G31.84. SCI was used as a diagnosis when the patients had subjective symptoms without objective clinical findings, using ICD code Z03.3. Patient demographics have been outlined in Table 1 and a flow diagram of the participants enrolled in the study can be seen in Figure 1. The presence of hypertension, hyperlipidemia, and diabetes were determined based on self-report/prior medical diagnosis and treatment for patients.

**Table 1:** Baseline demographics.

**Patient characteristics**	**Whole cohort** **(*n* = 520)**	**Alzheimer's disease** **(*n* = 150)**	**Mild cognitive impairment** **(*n* = 165)**	**Other disorders** **(*n* = 38)**	**Subjective cognitive impairment** **(*n* = 156)**	**Vascular dementia** **(*n* = 11)**
Age, mean (±SD)	62 (±9)	66 (±8)	63 (±8)	63 (±8)	57 (±8)	63 (±10)
Female, *n* (%)	306 (59)	92 (61)	84 (51)	19 (50)	104 (67)	7 (64)
MMSE, mean (±SD)	25 (±5)	22 (±5)	26 (±3)	23 (±6)	28 (±3)	23 (±3)
Diabetes, *n* (%)	39 (8)	7 (5)	15 (9)	3 (8)	11 (7)	3 (27)
Hyperlipidemia, *n* (%)	110 (21)	35 (23)	37 (22)	7 (18)	27 (17)	4 (36)
Hypertension, *n* (%)	187 (36)	61 (41)	76 (46)	9 (24)	34 (22)	7 (64)
Aβ 42, ng/L, median (IQR)	695 (482–998)	470 (385–574)	711 (489–975)	874 (704–1,230)	965 (744–1,175)	1,082 (856–1,322)
T-Tau, ng/l, median (IQR)	300 (303–489)	546 (364–760)	269 (183–415)	262 (198–423)	230 (174–318)	222 (192–294)
P-Tau, ng/l, median (IQR)	57 (42–81)	82 (64–108)	53 (36–70)	46 (37–57)	50 (38–62)	44 (33–61)
ALBR, median (IQR)	6 (5–8)	6 (4–8)	6 (5–8)	6 (6–8)	6 (5–7)	6 (4–10)
APOE ε2 carrier, *n* (%)	53 (10)	4 (3)	21 (13)	4 (11)	21 (14)	3 (27)
APOE ε3 carrier, *n* (%)	435 (84)	109 (73)	136 (82)	36 (95)	143 (92)	11 (100)
APOE ε4 carrier, *n* (%)	255 (49)	102 (68)	80 (49)	9 (24)	63 (40)	1 (9)
MRI Analysis						
CMB Prevalence, *n* (%)	101 (19)	42 (28)	33 (20)	7 (18)	14 (9)	5 (46)
Multiple CMBs, *n* (%)	53 (10)	25 (17)	22 (13)	1 (4)	3 (2)	2 (18)
Lobar CMBs, *n* (%)	85 (16)	37 (25)	29 (17)	5 (13)	10 (6)	4 (36)
Deep and Infratentorial CMBs, *n* (%)	45 (9)	17 (11)	18 (11)	3 (8)	5 (3)	2 (18)
WMH, mean score (±SD)	0.8 (±0.8)	1.0 (±0.8)	0.7 (±0.8)	0.8 (±0.7)	0.7 (±0.6)	1.9 (±1.2)
EPVS, mean score (±SD)	1.9 (±0.8)	1.9 (±0.9)	1.8 (±0.8)	2.0 (±0.9)	1.7 (±0.8)	2.4 (±0.9)
Large infarction, *n* (%)	16 (3)	4 (3)	4 (2)	2 (5)	1 (1)	5 (46)
Lacunar Infarction, *n* (%)	70 (14)	25 (17)	22 (13)	5 (13)	9 (6)	9 (82)
ICH, *n* (%)	7 (1)	3 (2)	2 (1)	0 (0)	1 (1)	1 (9)
Superficial Siderosis, *n* (%)	13 (3)	8 (5)	3 (2)	1 (3)	0 (0)	1 (9)

*ALBR, CSF/serum albumin ratio; CMB, Cerebral microbleeds; EPVS, Enlarged Perivascular Spaces; ICH, Intracerebral Hemorrhage; IQR, Interquartile range; T-tau, total tau; P-tau, Phosphorylated tau; WMH, White matter hyperintensities, rated according to the Fazekas scale*.

Informed consent was obtained for all patients according to the Declaration of Helsinki, and ethical approval was obtained from the regional ethical board in Stockholm, Sweden.

### MRI Protocol

Patients were scanned on three MRI scanners (Siemens Medical Systems, Erlangen, Germany) at the radiology department, Karolinska University Hospital, Huddinge. Axial SWI and/or T2^*^ sequences, as well as conventional MRI sequences, such as T1, T2, FLAIR (axial), and diffusion- weighted imaging (DWI), were obtained for all patients. Patients were randomly assigned to the different MRI scanners based on clinical availability, as well as the T2^*^ and SWI sequences. Furthermore, 155 patients were scanned on the 1.5T Siemens Magnetom Symphony, 212 patients on the 1.5T Siemens Magnetom Avanto, 153 patients were scanned on the Siemens Magnetom Trio 3.0T. In the whole cohort, the distribution of patients scanned on the 3T and with SWI sequences included were as follow: AD (3T: 27% and SWI: 19%), MCI (3T: 32% and SWI: 16%), SCI (3T: 28% and SWI: 17%), VaD (3T: 27% and SWI: 18%), and other dementias (3T: 32% and SWI: 24%).

### Image Analysis

All MRI images were jointly analyzed by a senior consultant neuroradiologist and an MD/PhD student with 3 years of training and experience in neuroradiology at the time of rating, with both being fully blinded to all patient data during rating.

CMBs were rated on axial T2^*^ and/or SWI according to the microbleed anatomical rating scale (Gregoire et al., 2009), with minor modifications to ensure increased accuracy of CMBs ratings as outlined previously (Shams et al., 2014). Modifications of the rating scale were as follows: CMBs were not rated as probable, and to reduce the number of CMB mimics, hypointensities in the globus pallidus, which may represent calcifications or physiologic iron deposits, were not rated, and similarly, images in which patients had a deep venous anomaly in the vicinity of CMBs were not rated as deep venous anomalies increase the risk of adjacent cavernomas, which can mimic a CMBs. Last, hemorrhagic sensitive sequences were analyzed together with T2-weighted images to better distinguish between vessels and flow voids, which also may mimic CMBs (Shams et al., 2014).

White matter hyperintensities were rated on axial FLAIR images according to the Fazekas scale (0 = none or single, 1 = punctate, 2 = early confluating, and 3 = confluating) (Fazekas et al., 1987) and the age-related white matter changes scale (Wahlund et al., 2001) (0 = none, 1 = punctate, 2 = early confluating, and 3 = confluating. Rated in the following brain regions: infratentorial, parieto-occipital, frontal, temporal, and the basal ganglia.). EPVSs were rated on an axial T2 according to the enlarged perivascular rating scale (0 = none, 1 = 1–10, 2 = 11–20, 3 = 21–40, and 4 = >40) (Maclullich et al., 2004; Doubal et al., 2010). EPVSs were defined as <3 mm in size and thus distinguished from lacunar infarctions that were defined as 3–15 mm in size, with cerebrospinal fluid (CSF) signal on FLAIR, T2 and T1. Cortical superficial siderosis was defined as a linear gyriform pattern of hypointense signal on T2^*^ and/or SWI (Feldman et al., 2008; Vernooij et al., 2009).

### CSF Analysis

Cerebrospinal fluid samples were obtained by lumbar puncture in a total of 399 patients and collected in 10 ml polypropylene tubes at the department of clinical chemistry, Karolinska University Hospital, Huddinge. All CSF samples were centrifuged within 2 h, at 1,900 g for 10 min and then frozen until analysis. A small amount of CSF was used for routine analysis of total cells, total protein, and glucose levels. Biomarkers were measured with a sandwich type enzyme linked immunosorbent assay; amyloid β 42 (Aβ42) was measured with Innotest β-Amyloid (1–42), total tau (T-tau) with Innotest hTau-Ag, and tau phosphorylated at threonine 181 (P-tau) with Innotest Phospho-tau(181P) (Innogenetics, Ghent, Belgium). The unit used for biomarkers is ng/L. Corresponding blood samples were collected at the same visit as the lumbar puncture to quantify CSF/serum albumin ratios (Tibbling et al., 1977). The team involved in CSF and blood analysis were unaware of the dementia diagnosis and MRI images.

### *APOE* Genotyping

The *APOE* genotyping was performed on all patients (*n* = 520) on coded genomic DNA samples. All analyses were performed at the department of clinical chemistry, Karolinska University Hospital. The team involved in the genotype analysis were unaware of the dementia diagnosis and the neuroimaging data.

### Statistics

Generalized linear models were used to determine the association between *APOE* genotype and MRI markers of SVD. Multiple binary logistic regression analyses were performed with the *APOE* genotype as an independent variable and dichotomized MRI markers as dependent variables. The *APOE* genotype was stratified into ε2 (such as, ε2/2, ε2/3, and ε2/4 alleles) and ε4 (ε4/4, ε4/3, and ε2/4 alleles). Separate analysis was done for ε4/4 when effect sizes were sufficient. *APOE* ε3/3 was used as a reference for all *APOE* genotypes in our regression models. White matter hyperintensities were dichotomized by separating high scores (2 and 3) from low scores (0 and 1) on the rating scales. Similarly, EPVSs were dichotomized by separating high scores (3 and 4) from low scores (0, 1, and 2) as per the enlarged perivascular scale. Multiple CMBs were defined as having more than 1 CMBs. Negative binomial regression analysis was performed to assess the association between *APOE* genotype and CMB topography. The number of CMBs in the different topographies was used as a dependent variable and the *APOE* genotype as an independent variable. Ordinal regression models were used with age related white matter changes and enlarged perivascular scores in different locations as dependent variables and the *APOE* genotype as an independent variable. All the above models were adjusted for age, gender, hypertension, hyperlipidemia, diabetes, and MRI field strength (and CMB sequence in the negative binomial regression analysis) due to the statistical significance of these markers in multivariable regression models. Linear regression models were used to study the cumulative effect of the presence of *APOE* ε4/4 or ε3/3 *and* the MRI marker in focus, on CSF biomarkers. Log transformed CSF biomarkers were put as dependent variables. The presence of *APOE* homozygote carriership *and* MRI marker was defined as a separate independent variable, and other independent adjusting variables in the model were: *APOE* genotype in focus, MRI marker of interest, age, gender, hypertension, hyperlipidemia, diabetes, dementia diagnosis, and MRI field strength. Due to the larger group size, statistical testing was focused on AD, MCI, and SCI. SPSS was used for the statistical analysis and *p* < 0.05 was set as the threshold of significance.

## Results

**Figure 2:**
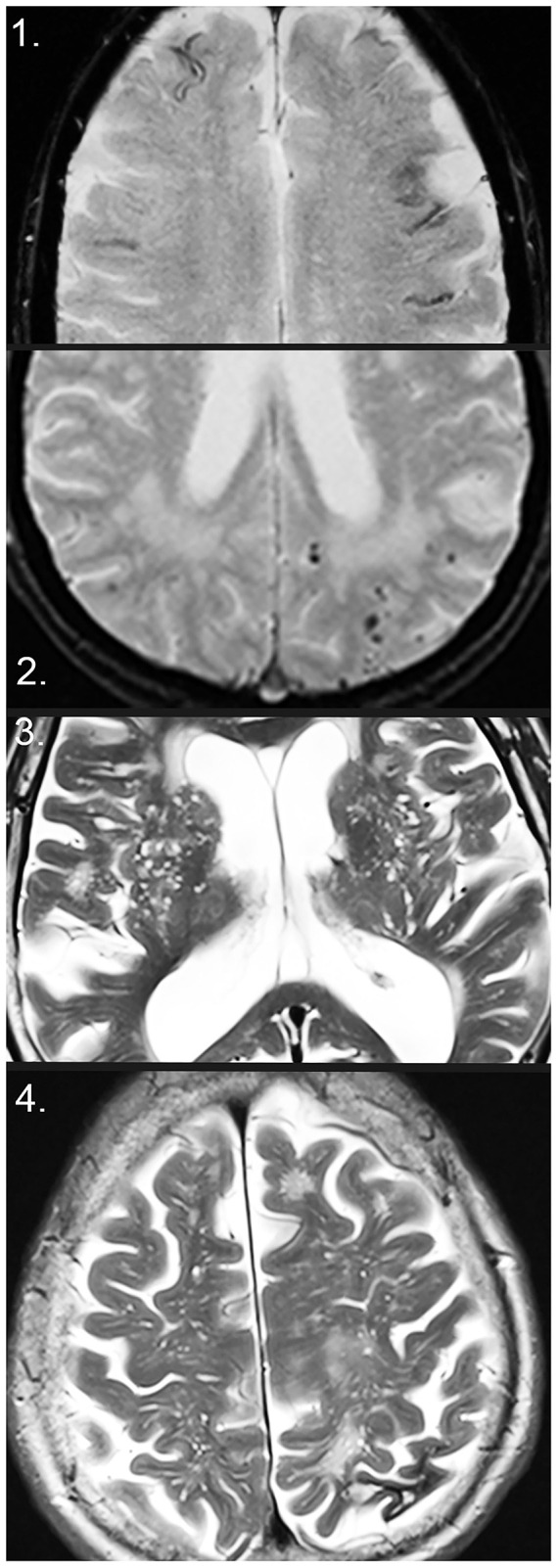
Markers of small vessel disease on MRI. (1) Disseminated superficial siderosis on T2*. (2) Cerebral microbleeds (CMBs), as well as white matter hyperintensities on T2*. (3) Enlarged perivascular spaces (EPVS) in the basal ganglia on T2. (4) EPVS in the centrum semiovale on T2 and intracerebral macrohemorrhage with local atrophy in the left parietal lobe.

Patient demographics are presented in Table 1 and imaging markers of SVD analyzed can be seen in Figure 2. In the whole cohort, APOE ε4, ε4/4, and ε2 carriers showed a higher number of CMBs (*p* < 0.05). A higher number of lacunar infarcts were seen in *APOE* ε4/4 carriers with MCI (*p* = 0.01) and *APOE* ε2 carriers with SCI (*p* = 0.01). Table 2 shows the odds ratios (*OR*s) for the SVD markers on MRI depending on the *APOE* genotype. Only two patients with SCI had *APOE* ε2/2 and both had the lowest dichotomized white matter hyperintensity and enlarged perivascular score, had no CMBs, large or lacunar infarctions, intracerebral hemorrhage, or cortical superficial siderosis.

**Table 2:** Odds ratios (*OR*s) and 95% confidence interval (*CI*) for markers of small vessel disease by *APOE* alleles.

	**Odds ratio (95 CI)**									
	**CMBs**	**Multiple CMBs**	**Lobar CMBs**	**Deep and infratentorial CMBs**	**WMH**	**Lacunar infarction**	**EPVS**	**Siderosis**	**Large infarction**	**ICH**
***APOE*** **Genotype whole cohort (*****n*** **=** **520)**										
ε2	0.9 (0.4–2.0)	18.8 (1.6–22.0)^**a**^	0.9 (0.4–2.3)	1.9 (0.7–5.4)	2.3 (0.9–5.7)^**d**^	2.1 (0.9–5.0)^**d**^	1.1 (0.5–2.3)	2.0 (0.3–12.1)	0.8 (0.2–4.0)	4.8 (0.6–36.4)
ε4	1.3 (0.7–2.1)	3.0 (1.1–8.0)^**a**^	1.3 (0.7–2.2)	1.4 (0.7–3.0)	1.3 (0.7–2.6)	1.2 (0.7–2.2)	1.0 (0.6–1.7)	0.6 (0.2–2.7)	0.3 (0.1–1.1)^**c**^	1.6 (0.3–10.0)
ε4/4	1.3 (0.6–2.8)	8.2 (1.7–11.2)^**a**^	1.4 (0.6–3.1)	1.2 (0.4–3.8)	0.5 (0.2–1.7)	1.7 (0.7–3.9)	0.9 (0.5–1.9)	2.8 (0.6–12.8)	0.3 (0.0–2.2)	1.9 (0.2–22.8)
**AD (*****n*** **=** **150)**									-	-
ε2	1.7 (0.2–18.2)		1.7 (0.2–18.2)	5.0 (0.1–22.7)	-	-	1.0 (0.1–16.4)	-		
ε4	1.5 (0.6–4.0)	2.0 (0.3–13.1)	1.1 (0.4–2.9)	5.2 (0.9–27.5)^**b**^	1.2 (0.4–4.0)	1.3 (0.4–4.2)	1.4 (0.5–3.8)	0.4 (0.0–3.7)	0.6 (0.1–5.0)	0.2 (0.0–5.0)
ε4/4	1.8 (0.5–5.8)	2.0 (0.2–20.2)	1.3 (0.4–4.3)	2.8 (0.4–18.1)	0.6 (0.1–3.5)	1.9 (0.4–9.3)	2.8 (0.8–9.8)	1.7 (0.2–15.4)	-	0.7 (0.0–13.3)
**MCI (*****n*** **=** **156)**									-	-
ε2	1.0 (0.3–3.9)	0.2 (0.0–57.3)	0.9 (0.2–4.2)	1.3 (0.3–5.3)	3.5 (0.7–18.8)	1.7 (0.3–9.1)	1.3 (0.4–4.2)	-		-
ε4	1.2 (0.5–3.0)	0.8 (0.1–5.8)	1.3 (0.5–3.4)	0.5 (0.1–1.7)	1.8 (0.5–6.4)	1.8 (0.5–6.0)	0.8 (0.3–1.7)	0.6 (0.0–10.1)	0.4 (0.0–5.0)	-
ε4/4	1.1 (0.3–4.5)	1.0 (0.1–14.9)	1.2 (0.3–5.0)	0.4 (0.0–3.6)	1.0 (0.1–7.3)	5.4 (1.2–25.3)^**a**^	0.4 (0.1–1.6)	3.0 (0.1–85.3)	1.5 (0.1–19.0)	
**SCI (*****n*** **=** **165)**		-		-					-	-
ε2	0.4 (0.0–3.8)	-	0.8 (0.1–8.1)		0.8 (0.0–17.1)	10.7 (1.7–69.6)^**a**^	0.2 (0.0–1.9)	-	-	-
ε4	0.7 (0.2–2.6)		0.9 (0.2–4.1)	1.1 (0.1–7.5)	2.2 (0.3–18.6)	0.5 (0.1–2.6)	1.0 (0.4–2.6)	-		

*Multiple binary logistic regression analyses were performed with the APOE genotype as an independent variable and the dichotomized MRI markers as dependent variables. APOE ε3/3 was used as a reference for all APOE genotypes. The model was controlled for age, gender, hypertension, hyperlipidemia, diabetes, and MRI field strength. APOE alleles were excluded from analysis if effect sizes were considered too small (e.g., no MRI marker); similarly “-” indicates too small effect size to be computed. Multiple CMBs=over 1 CMB. EPVS was dichotomized into 0 (score 0, 1, and 2), 1 (score 3 and 4). WMH are rated according to the Fazekas scale and dichotomized as 0 (score 0 and 1), 1 (score 2 and 3). AD, Alzheimer's disease; CMB, Cerebral microbleeds; EPVS, Enlarged Perivascular Spaces; ICH, Intracerebral Hemorrhage; MCI, Mild cognitive Impairment; SCI, Subjective cognitive impairment; WMH, White matter hyperintensities. ^a^p < 0.05; ^b^p = 0.05–0.06; ^c^p = 0.07–0.08; ^d^p = 0.08–0.09*.

### *APOE* Alleles and Cerebral Microbleeds

The association between the *APOE* allele and the number of MRI markers of SVD can be seen in Table 3.

**Table 3:** Regression coefficients for number/score of MRI markers by *APOE* alleles.

	**Regression coefficient B**													
**APOE genotype**	**all regions**	**Lobar**	**Deep and infratentorial**	**Occipital**	**Parietal**	**Temporal**	**Frontal**	**Parietooccipital**	**Frontal**	**Temporal**	**Basal ganglia**	**Infratentorial**	**centrum semiovale**	**Basal ganglia**
Whole cohort (n=520)														
ε2	−0.4^c^	−0.6^b^	0.6^b^	−0.7^b^	−0.7	0.0	0.4	0.5	0.8^b^	0.9^c^	0.4	0.9^e^	−0.25	0.2
ε3	−0.7^a^	−1.0	0.8^b^	−1.1^a^	−1.4^a^	−1.1^a^	−1.2^a^	0.4^e^	0.3	0.4	0.2	0.5	0.4^e^	0.3
ε3/3	−1.1^a^	−1.7^a^	−0.0	−3.5^a^	−1.9^a^	−1.8^a^	−0.5^b^	−0.3	−0.0	−0.1	0.0	−0.1	0.1	0.5^b^
ε4	1.0^a^	1.5^a^	−0.4^d^	2.5^a^	1.7^a^	1.3^a^	0.6^a^	0.1	−0.2	−0.3	−0.11	−0.3	−0.1	−0.6^b^
ε4/4	0.9^a^	1.3^a^	−0.5	1.4^a^	1.7^a^	1.4^a^	1.4^a^	−0.4	−0.5^f^	−0.5	−0.3	−0.7	−0.4	−0.2
AD (n=150)														
ε2	−0.4	0.1	−0.9	−0.8	0.3	1.2	0.6	−0.7	−0.5	–	0.1	–	−0.3	−0.8
ε3	−0.5^c^	−1.1^b^	0.4	−0.6	−1.8^b^	−1.1^b^	−1.1^b^	0.2	0.4	0.1	0.2	0.3	−0.1	0.0
ε3/3	−1.2^a^	−2.4^a^	0.4	−3.1^a^	−3.0^a^	−2.3^b^	−3.0^a^	−0.5	−0.3	0.1	−0.1	0.4	−0.2	0.4
ε4	1.1^a^	1.8^a^	−0.3	2.6^a^	2.3^a^	1.3^b^	1.4^b^	0.6	0.2	−0.0	0.2	−0.3	0.2	−0.4
ε4/4	0.7^b^	1.2^a^	−0.3	0.8^b^	2.0^a^	1.4^b^	1.2^b^	−0.2	−0.3	−0.1	−0.4	−0.4	0.2	0.1
MCI (n=156)														
ε2	−0.6^e^	−0.7^d^	−0.1	−0.7	−0.3	−0.7	−1.4^f^	0.5	0.8^f^	1.6^c^	0.3	1.8^b^	0.6	0.4
ε3	−0.8^b^	−1.0^b^	1.8 ^b^	−1.5^a^	−0.9^c^	−1.2^b^	−1.5^a^	0.1	−0.1	0.9	−0.1	-	0.6	0.2
ε3/3	−1.0^a^	−1.9^a^	0.7 ^b^	−31.0	−1.7^b^	−1.5^b^	−0.4	−0.5	−0.1	−0.2	0.3	0.1	0.2	0.6
ε4	0.8^a^	1.5^a^	−0.91^b^	2.7^a^	1.2^b^	1.3^b^	0.6	0.2	−0.1	−0.6	−0.6	−1.4^g^	−0.4	−0.7^b^
ε4/4	1.4^a^	1.5^a^	−1.3^f^	2.1^a^	1.4^b^	1.6^b^	2.0^a^	−0.0	−0.3	-	0.3	-	−1.1^b^	−0.2
SCI (n=165)														
ε2	−2.7^b^	−2.6^c^	−30.1	-	-	-	-	−0.1	0.3	1.0	0.1	−0.5	−0.9^g^	0.0
ε3/3	−1.1^b^	−1.1^f^	−0.9	−1.3	−0.9	-	-	0.1	0.4	0.3	−0.0	−2.2	0.3	0.7
ε4	1.7^b^	1.7 ^b^	1.8 ^b^	1.9 ^b^	1.1	–	-	−0.3	−0.6	−1.2	−0.0	4.7	−0.1	−0.9^g^

*A negative binomial regression was performed with number of CMBs as a dependent variable, and the APOE genotype as an independent variable. The model was controlled for: age, gender, hypertension, hyperlipidemia, diabetes, and MRI field strength (and CMB sequence, when analyzing CMBs). Additionally, ordinal regression analyses were performed with the ARWMC and EPVS scores in different brain regions. AD= Alzheimer's disease; ARWMC, Age related white matter changes; CMB, cerebral microbleeds; EPVS, Enlarged perivascular spaces; MCI, Mild cognitive impairment; SCI, subjective cognitive impairment. ^a^p < 0.001; ^b^p < 0.05; ^c^p = 0.05–0.06;^d^p = 0.06–0.07;^e^p = 0.07–0.08; ^f^p = 0.08–0.09; ^g^p = 0.09–0.1*.

#### APOE ε4

In the whole cohort, *APOE* ε4 and ε4/4 carriers showed a higher number of CMBs, especially in lobar brain regions (*p* < 0.001). A separate analysis of each brain lobe in ε4 and ε4/4 carriers showed a significantly higher number of CMBs (*p* < 0.001). Patients with AD and MCI who were *APOE* ε4 carriers had a higher number of CMBs in the lobar brain region. Patients with SCI who were *APOE* ε4 carriers had a higher number of CMBs in lobar, deep, infratentorial, and occipital brain regions (*p* < 0.05).

#### APOE ε2 and ε3

The ε2, ε3, and ε3/3 carriers had a lower number of CMBs (*p* < 0.001). Topographical analysis showed that CMBs were lower in the brain lobes in ε2 and ε3/3 carriers, lower in the occipital lobe in ε2, ε3, and ε3/3 carriers, and lower in the parietal lobe in ε3 and ε3/3 carriers (*p* < 0.001). The number of CMBs in deep and infratentorial regions were higher in *APOE* ε2 and ε3 carriers (*p* < 0.05). This pattern was also seen when looking into the separate diagnostic groups. Patients with AD or MCI who were ε3 carriers had a lower number of CMBs in lobar regions (*p* < 0.001).

### *APOE* Alleles, MRI Markers of SVD, and CSF Biomarkers

Table 4 shows the relationship between CSF biomarkers and SVD markers in *APOE* carriers.

**Table 4:** The *APOE* genotype, MRI markers, and associations with CSF biomarkers.

	**Regression Coefficient B (*****n*** **=** **399)**										
		**CMB**		**WMH**		**Lacunar Infarcts**		**EPVS**		**Siderosis**	
**Whole Cohort**	**APOE**	**ε3/3**	**ε4/4**	**ε3/3**	**ε4/4**	**ε3/3**	**ε4/4**	**ε3/3**	**ε4/4**	**ε3/3**	**ε4/4**
	Aβ 42	0.02	−0.20^a^	0.03	−0.14^a^	0.05	−0.18^a^	0.00	−0.17^a^	0.00	−0.27^b^
	T-tau	−0.01	0.09	−0.14^b^	0.08	0.03	0.00	0.03	0.07	0.16	0.02
	P-tau	−0.03	0.09	−0.16^b^	−0.03	−0.01	−0.02	0.01	0.10^b^	0.15	0.05
	ALBR	−0.04	0.02	−0.03	−0.05	0.10	0.04	0.02	0.03	−0.06	0.07
**Alzheimer's disease**											
	Aβ 42	−0.04	−0.15^b^	−0.02	−0.33^a^	0.01	−0.14^b^	−0.03	−0.12^b^	−0.01	−0.20^b^
	T-tau	−0.05	−0.03	−0.06	−0.16	−0.01	−0.18^g^	−0.20^d^	0.05	0.05	−0.09
	P-tau	−0.07	0.03	−0.07	−0.10	−0.02	−0.09	−0.17^d^	0.10	−0.02	−0.00
	ALBR	−0.06	0.00	0.02	−0.06	0.01	0.00	0.04	0.05	0.00	0.14
**Mild cognitive impairment**											
	Aβ 42	−0.09	−0.29^a^	−0.04	−0.32^b^	0.15	−0.23^b^	0.11^b^	−0.24^b^	−0.19	−0.39^b^
	T-tau	−0.06	0.25^b^	−0.18	0.29^d^	−0.19	0.27^b^	−0.04	0.21^c^	−0.04	0.21
	P-tau	−0.05	0.15	−0.28^b^	0.08	−0.21^c^	0.18^b^	−0.07	0.09	−0.07	0.15
	ALBR	−0.09	0.04	−0.10	−0.02	−0.03	0.02	−0.03	−0.09	−0.16	−0.08
**Subjective cognitive impairment**											
	Aβ 42	0.18^b^	−0.04	0.10	−0.18^c^	0.07	0.09	0.14^b^	−0.09^d^	-	-
	T-tau	−0.02	0.03	−0.17	0.20	0.06	0.07	0.01	0.01	-	-
	P-tau	−0.00	0.03	−0.04	0.12	0.03	0.06	−0.02	−0.03	-	-
	ALBR	0.00	−0.01	−0.12	0.07	0.04	−0.12^f^	0.01	−0.80	-	-

*Linear regression analysis was performed with the CSF biomarker as a dependent variable and the presence of MRI marker and the APOE genotype of interest entered as one independent variable. The model was controlled for: the APOE genotype of interest, the MRI marker in focus, age, gender, dementia diagnosis, hypertension, hyperlipidemia, diabetes, and MRI field strength. All patients with CSF biomarkers were included in this analysis (n = 399). CMBs, lacunar infarcts and siderosis indicates the presence of these markers. WMH and EPVS are dichotomized values. In subjective cognitive impairment analysis for ε3/3 was performed as before, however, due to small effect sized, analysis was performed for ε4 carriers. ALBR, CSF/serum albumin ratio; CMB, Cerebral Microbleeds; EPVS, Enlarged perivascular spaces; WMH, White matter hyperintensities. ^a^p < 0.001;^b^p < 0.05; ^c^p = 0.05–0.06;^d^p = 0.06–0.07; ^d^p = 0.07–0.08; ^f^p = 0.08–0.09; ^g^p = 0.09–0.10*.

#### APOE ε4

In the whole cohort, there was a negative association between the level of CSF Aβ42 and the number of CMBs, white matter hyperintensities, lacunar infarcts, enlarged perivascular spaces, and cortical superficial siderosis in *APOE* ε4/4 carriers (*p* < 0.001). This held true in AD as well as MCI. In the whole cohort, an association was seen in *APOE* ε4/4 carriers with the higher levels of CSF P-Tau and a higher enlarged perivascular space score (*p* < 0.05). *APOE* ε4/4 carriers with MCI demonstrated an association between higher T-tau and P-tau levels with lacunar infarctions and higher T-tau with CMBs (*p* < 0.05).

#### APOE ε3

Apolipoprotein E ε3/3 carriers showed lower levels of CSF T-tau and P-tau with increasing white matter hyperintensity (*p* < 0.05). In *APOE* ε3/3, a lower level of P-tau was observed with white matter hyperintensity (*p* < 0.05). *APOE* ε3/3 carriers with SCI showed a higher CSF Aβ42 concentration with CMBs and EPVSs (*p* < 0.05).

### *APOE* Alleles and MRI Markers of SVD in Vascular Dementia

All patients with vascular dementia were *APOE* ε3 carriers (7 patients homozygous for the ε3 allele and 4 patients being heterozygous for the ε3 allele). Comparing these two groups, we found that among ε3 carriers, 2/4 had CMBs, 3/4 had high white matter hyperintensity score, 2/4 had high enlarged perivascular space score, 3/4 had lacunar infarcts, 2/4 had a large infarction, no one had intracerebral hemorrhage, and 1/4 had cortical superficial siderosis. In patients homozygous for the ε3 allele, 3/7 had CMBs, 3/7 had high white matter hyperintensity score, 2/7 had high enlarged perivascular space score, 6/7 had lacunar infarcts, 3/7 had large infarcts, 1/7 had a large bleeding, and no one had siderosis.

## Discussion

We show that *APOE* ε4 carriers have more pronounced SVD MRI markers associated with amyloid pathology, whereas ε3 and ε2 carriers demonstrate MRI markers related with hypertensive arteriopathy. CSF profiles with the presence of SVD in *APOE* ε4 carriers indicate the importance of SVD in the clinical continuum of AD pathology, from MCI to AD.

Previous studies investigating markers of SVD and *APOE* genotype in dementia are scarce. In healthy populations, a higher number of lobar CMBs with the *APOE* ε4 allele, compared with carriers of ε3/3, have been seen (Poels et al., 2012), in line with our results. The association between possession of the *APOE* ε4 or ε2 genotype and lobar CMBs has also been shown in a stroke population (Kim et al., 2013). However, in the Framingham study, no relationship between the *APOE* allele and CMBs was found (Jeerakathil et al., 2004).

White matter hyperintensities, studied in general populations, have been shown to increase with *APOE* ε4, ε4/4, and ε2 (Schilling et al., 2013). Increased white matter hyperintensity volume in the parietal lobe has been shown to predict incident AD and increased parietal white matter hyperintensity volume has been shown to be linked with *APOE* ε4 (Brickman et al., 2012, 2014). We demonstrated an association between *APOE* ε2 and higher white matter hyperintensity burden in frontal brain regions. No other relationships between white matter hyperintensity burden, globally or in different brain regions, and the *APOE* genotype was found. No relationship between *APOE* and lacunar infarcts has been shown (Kim et al., 2013).

To the best of our knowledge, EPVSs and *APOE* genotype in dementia have not been investigated previously. Cortical superficial siderosis has been reported to be associated with a *APOE* ε4 in a memory clinic population (Zonneveld et al., 2014), but this was not demonstrated in our cohort. Intracerebral hemorrhage has been associated with *APOE* ε4 (Brickman et al., 2014), however, no such relationship was seen in our cohort.

The association between SVD with *APOE* ε2 are of special interest, as *APOE* ε2, in contrast to the ε4 allele, is considered to be a protective allele with lower risk of AD related neurodegeneration (Suri et al., 2013). The presence of the *APOE* ε2 allele has been shown to decrease the risk for AD by a factor of 4 (Corder et al., 1994; Suri et al., 2013). When looking at the *APOE* ε2 allele, we could see a lower number of lobar CMBs, and a higher number of deep and infratentorial CMBs. The pattern of CMBs seen in *APOE* ε2 carriers support that deep and infratentorial CMBs are of a different pathogenesis than lobar CMBs, and presumably have little implication in the pathophysiology of AD. However, the positive association between cortical superficial siderosis with *APOE* ε2, but not *APOE* ε4, shown in a study in cerebral amyloid angiopathy patients is interesting and may suggest that the underlying pathophysiologic mechanism giving rise to cortical superficial siderosis differs from that giving rise to CMBs in cerebral amyloid angiopathy (Shoamanesh et al., 2014). Moreover, a higher white matter hyperintensity burden was seen in frontal and infratentorial brain regions, in the whole cohort and MCI, respectively, with *APOE* ε2. A higher number of lacunar infarcts were seen in *APOE* ε2 carriers in SCI. Our results imply that *APOE* ε2 is associated with hypertensive arteriopathy, as reflected through the association with deep and infratentorial CMBs, white matter hyperintensity, and lacunar infarcts, and thus hypertensive arteriopathy may cause cortical superficial siderosis and in part explain the increased association of siderosis with *APOE* ε2.

The *APOE* genotype, imaging markers of SVD (such as, CMBs, white matter hyperintensities, and lacunar infarcts), and CSF biomarkers in dementia have previously been investigated and have shown lower CSF Aβ42 levels with the presence of CMBs and white matter hyperintensities in *APOE* ε4 carriers, reflecting increased deposition of Aβ42 in the brain parenchyma (Shoamanesh et al., 2014).

We included additional markers of SVD and demonstrated decreased Aβ42 levels with all included SVD markers in *APOE* ε4/4 carriers. Moreover, we found higher T-tau and P-tau levels with some SVD markers in *APOE* ε4/4 carriers in patients with MCI, reflecting the importance of SVD markers in an early stage of dementia, possibly contributing to the dementia pathophysiology. As this relationship was only seen in MCI, it may imply that SVD markers in the early stages of dementia contribute to neuronal damage and facilitates the formation of neurofibrillary tangles as represented by T-tau and P-tau, respectively, ultimately contributing to the final picture of AD. Further on, this implies that the pathophysiology of dementia may partly be mediated through SVD. Patients with SCI and *APOE* ε4, however, demonstrated minor association between CSF biomarkers and SVD markers, although tendencies toward lower Aβ42 levels with EPVSs and white matter hyperintensities were seen. This may be explained by the fact that SCI represents a heterogeneous group of individuals with cognitive complaints, and that only heterozygous carriers for the ε4 allele were analyzed due to the scarcity of homozygous ε4 carriers in SCI.

To further investigate our hypothesis on the importance of SVD in the early stages of dementia, prospective, longitudinal studies on patients with SCI and MCI should be conducted to see if patients with SVD develop dementia more rapidly.

The stratification of SVD markers in hypertensive arteriopathy with *APOE* ε3 and ε2, and an amyloid-based pattern with the *APOE* ε4 genotype, is of interest. In our cohort, all patients with vascular dementia were ε3 carriers and had an overweight of vascular SVD markers, with high baseline CSF Aβ42 levels. However, the synergism between vascular and amyloid pathology, and their overlap in MRI SVD expression is of importance to keep in mind. Amyloid has been shown to impair vessel function and lead to small vessel ischemia (Kester et al., 2014), which may lead to the development of markers caused by vascular pathology, such as lacunar infarctions, cerebral microinfarcts, and white matter hyperintensities. Furthermore, the association of *APOE* ε2 with markers of hypertensive arteriopathy may not be direct, but rather due to the fact that *APOE* ε2 carriers tend to have a lower amyloid burden and thus require a greater severity of hypertensive SVD for cognitive impairment to develop. The same alternate explanation may hold true for *APOE* ε3 carriers, where an association with hypertensive arteriopathy was seen.

The strengths of our study include a large cohort, with patients undergoing thorough dementia investigation as well as neuroradiological analysis. Limitations include a small cohort size when analyzing the separate diagnostic groups, such as vascular dementia, and analyses were excluded if effect sizes in the separate diagnoses were too small. Another limitation is that the dementia diagnosis was set after considering both the clinical and radiological picture, which may have affected the final designation of diagnosis.

In conclusion, our study emphasizes the possible importance of SVD in a continuum of cognitive impairment. *APOE* ε4 is mainly associated with markers of the amyloid pathology, whereas *APOE* ε3 and ε2 demonstrate associations with hypertensive arteriopathy.

## Data Availability Statement

The raw data supporting the conclusions of this article will be made available by the authors, without undue reservation.

## Ethics Statement

The studies involving human participants were reviewed and approved by the regional ethical board in Stockholm, Sweden. The patients/participants provided their written informed consent to participate in this study.

## Author Contributions

MS: collection of data, establishing database, statistical analysis, and drafting of manuscript. SS: study concept and design, data acquisition, establishing database, and image analysis. JM and LC: image analysis. TG: data acquisition and power calculations for the KIDS. MK: project supervision, study design, critical analysis, and revision of the manuscript. MW: project supervision, critical analysis, and revision of the manuscript. EW: critical analysis and revision of the manuscript. PA, MKW, and L-OW: project supervision, study and planning, patient inclusion and recruitment, and as well as clinical and MRI logistics. All authors designed and conceptualized the study, engaged in data analysis and subsequent critical analysis, and revision of the manuscript. All authors contributed to the article and approved the submitted version.

## Funding

This study is funded by the Stockholm County Council, Karolinska Institutet, and the Swedish Dementia Association.

## Conflict of Interest

The authors declare that the research was conducted in the absence of any commercial or financial relationships that could be construed as a potential conflict of interest.

## Publisher's Note

All claims expressed in this article are solely those of the authors and do not necessarily represent those of their affiliated organizations, or those of the publisher, the editors and the reviewers. Any product that may be evaluated in this article, or claim that may be made by its manufacturer, is not guaranteed or endorsed by the publisher.

## Abbreviations

MRI: Magnetic resonance imaging
FLAIR: Fluid attenuated inversion recovery
KIDS: Karolinska imaging dementia study
SVD: Small vessel disease
APOE: Apolipoprotein E
CSF: Cerebrospinal fluid
AD: Alzheimer's disease
MCI: Mild cognitive impairment
SCI: Subjective cognitive impairment.

## References

[B1] Braffman B. H. Zimmerman R. A. Trojanowski J. Q. Gonatas N. K. Hickey W. F. Schlaepfer W. W. (1988). Brain MR: pathologic correlation with gross and histopathology. 1. Lacunar infarction and Virchow-Robin spaces. AJR Am. J. Roentgenol. 151, 551–558. 10.2214/ajr.151.3.551 3261517

[B2] Braun H. Schreiber S. (2013). Microbleeds in cerebral small vessel disease. Lancet Neurol. 12, 735–736. 10.1016/S1474-4422(13)70148-0 23867194

[B3] Brickman A. M. Honig L. S. Scarmeas N. Tatarina O. Sanders L. Albert M. S.. (2008). Measuring cerebral atrophy and white matter hyperintensity burden to predict the rate of cognitive decline in Alzheimer disease. Arch. Neurol. 65, 1202–1208. 10.1001/archneur.65.9.1202 18779424 PMC2629007

[B4] Brickman A. M. Provenzano F. A. Muraskin J. Manly J. J. Blum S. Apa Z.. (2012). Regional white matter hyperintensity volume, not hippocampal atrophy, predicts incident Alzheimer disease in the community. Arch. Neurol. 69, 1621–1627. 10.1001/archneurol.2012.1527 22945686 PMC3597387

[B5] Brickman A. M. Schupf N. Manly J. J. Stern Y. Luchsinger J. A. Provenzano F. A.. (2014). APOE ε4 and risk for Alzheimer's disease: Do regionally distributed white matter hyperintensities play a role? Alzheimers Dement J Alzheimers Assoc 10, 619–629. 10.1016/j.jalz.2014.07.155 25304991 PMC4252241

[B6] Brun A. Englund E. (1986). A white matter disorder in dementia of the Alzheimer type: a pathoanatomical study. Ann. Neurol. 19, 253–262. 10.1002/ana.410190306 3963770

[B7] Charidimou A. Jäger R. H. Fox Z. Peeters A. Vandermeeren Y. Laloux P.. (2013). Prevalence and mechanisms of cortical superficial siderosis in cerebral amyloid angiopathy. Neurology 81, 626–632. 10.1212/WNL.0b013e3182a08f2c 23864315

[B8] Corder E. H. Saunders A. M. Risch N. J. Strittmatter W. J. Schmechel D. E. Gaskell P. C.. (1994). Protective effect of apolipoprotein E type 2 allele for late onset Alzheimer disease. Nat. Genet. 7, 180–184. 10.1038/ng0694-180 7920638

[B9] Cordonnier C. van der Flier W. M. (2011). Brain microbleeds and Alzheimer's disease: innocent observation or key player? Brain. 10.1017/CBO9780511974892.016 21257651

[B10] Cordonnier C. van der Flier W. M. Sluimer J. D. Leys D. Barkhof F. Scheltens P. (2006). Prevalence and severity of microbleeds in a memory clinic setting. Neurology 66, 1356–1360. 10.1212/01.wnl.0000210535.20297.ae 16682667

[B11] Doubal F. N. MacLullich A. M. J. Ferguson K. J. Dennis M. S. Wardlaw J. M. (2010). Enlarged perivascular spaces on MRI are a feature of cerebral small vessel disease. Stroke J. Cereb. Circ. 41, 450–454. 10.1161/STROKEAHA.109.564914 20056930

[B12] Fazekas F. Chawluk J. B. Alavi A. Hurtig H. I. Zimmerman R. A. (1987). MR signal abnormalities at 1.5 T in Alzheimer's dementia and normal aging. AJR Am. J. Roentgenol. 149, 351–356. 10.2214/ajr.149.2.351 3496763

[B13] Feldman H. H. Maia L. F. Mackenzie I. R. A. Forster B. B. Martzke J. Woolfenden A. (2008). Superficial siderosis: a potential diagnostic marker of cerebral amyloid angiopathy in Alzheimer disease. Stroke J. Cereb. Circ. 39, 2894–2897. 10.1161/STROKEAHA.107.510826 18635858

[B14] Greenberg S. M. Rebeck G. W. Vonsattel J. P. Gomez-Isla T. Hyman B. T. (1995). Apolipoprotein E epsilon 4 and cerebral hemorrhage associated with amyloid angiopathy. Ann. Neurol. 38, 254–259. 10.1002/ana.410380219 7654074

[B15] Gregoire S. M. Chaudhary U. J. Brown M. M. Yousry T. A. Kallis C. Jäger H. R.. (2009). The Microbleed Anatomical Rating Scale (MARS): reliability of a tool to map brain microbleeds. Neurology 73, 1759–1766. 10.1212/WNL.0b013e3181c34a7d 19933977

[B16] Jeerakathil T. Wolf P. A. Beiser A. Hald J. K. Au R. Kase C. S.. (2004). Cerebral microbleeds: prevalence and associations with cardiovascular risk factors in the Framingham Study. Stroke J. Cereb Circ. 35, 1831–1835. 10.1161/01.STR.0000131809.35202.1b 15155954

[B17] Jellinger K. A. (2002). Alzheimer disease and cerebrovascular pathology: an update. J. Neural. Transm. 109, 813–836. 10.1007/s007020200068 12111471

[B18] Kester M. I. Goos J. D. C. Teunissen C. E. Benedictus M. R. Bouwman F. H. Wattjes M. P.. (2014). Associations between cerebral small-vessel disease and Alzheimer disease pathology as measured by cerebrospinal fluid biomarkers. JAMA Neurol. 71, 855–862. 10.1001/jamaneurol.2014.754 24818585

[B19] Kim H. J. Ye B. S. Yoon C. W. Cho H. Noh Y. Kim G. H.. (2013). Effects of APOE ε4 on brain amyloid, lacunar infarcts, and white matter lesions: a study among patients with subcortical vascular cognitive impairment. Neurobiol. Aging 34, 2482–2487. 10.1016/j.neurobiolaging.2013.05.009 23769398

[B20] Maclullich A. M. J. Wardlaw J. M. Ferguson K. J. Starr J. M. Seckl J. R. Deary I. J. (2004). Enlarged perivascular spaces are associated with cognitive function in healthy elderly men. J. Neurol. Neurosurg. Psychiatry 75, 1519–1523. 10.1136/jnnp.2003.030858 15489380 PMC1738797

[B21] Pantoni L. (2010). Cerebral small vessel disease: from pathogenesis and clinical characteristics to therapeutic challenges. Lancet Neurol. 9, 689–701. 10.1016/S1474-4422(10)70104-6 20610345

[B22] Poels M. M. F. Ikram M. A. van der Lugt A. Hofman A. Niessen W. J. Krestin G. P.. (2012). Cerebral microbleeds are associated with worse cognitive function: the Rotterdam Scan Study. Neurology 78, 326–333. 10.1212/WNL.0b013e3182452928 22262748

[B23] Roher A. E. Kuo Y. M. Esh C. Knebel C. Weiss N. Kalback W.. (2003). Cortical and Leptomeningeal Cerebrovascular Amyloid and White Matter Pathology in Alzheimer's Disease. Mol. Med. 9, 112–122. 10.1007/BF03402043 12865947 PMC1430731

[B24] Schilling S. DeStefano A. L. Sachdev P. S. Choi S. H. Mather K. A. DeCarli C. D.. (2013). APOE genotype and MRI markers of cerebrovascular disease: systematic review and meta-analysis. Neurology 81, 292–300. 10.1212/WNL.0b013e31829bfda4 23858411 PMC3770168

[B25] Shams S. Martola J. Granberg T. Li X. Shams M. Fereshtehnejad S. M.. (2014). Cerebral microbleeds: different prevalence, topography, and risk factors depending on dementia diagnosis—the karolinska imaging dementia study. Am J Neuroradiol. 10.3174/ajnr.A4176 25523590 PMC7964321

[B26] Shoamanesh A. Martinez-Ramirez S. Oliveira-Filho J. Reijmer Y. Falcone G. J. Ayres A.. (2014). Interrelationship of superficial siderosis and microbleeds in cerebral amyloid angiopathy. Neurology 83, 1838–1843. 10.1212/WNL.0000000000000984 25320098 PMC4240429

[B27] Smith E. E. Greenberg S. M. (2009). Beta-amyloid, blood vessels, and brain function. Stroke J. Cereb. Circ. 40, 2601–2606. 10.1161/STROKEAHA.108.536839 19443808 PMC2704252

[B28] Suri S. Heise V. Trachtenberg A. J. Mackay C. E. (2013). The forgotten APOE allele: a review of the evidence and suggested mechanisms for the protective effect of APOE ε2. Neurosci. Biobehav. Rev. 37, 2878–2886. 10.1016/j.neubiorev.2013.10.010 24183852

[B29] Tibbling G. Link H. Ohman S. (1977). Principles of albumin and IgG analyses in neurological disorders. I. Establishment of reference values. Scand. J. Clin. Lab. Invest. 37, 385–390. 10.3109/00365517709091496 337459

[B30] Verghese P. B. Castellano J. M. Holtzman D. M. (2011). Apolipoprotein E in Alzheimer's disease and other neurological disorders. Lancet Neurol. 10, 241–252. 10.1016/S1474-4422(10)70325-2 21349439 PMC3132088

[B31] Vernooij M. W. Ikram M. A. Hofman A. Krestin G. P. Breteler M. M. B. van der Lugt A. (2009). Superficial siderosis in the general population. Neurology 73, 202–205. 10.1212/WNL.0b013e3181ae7c5e 19620607

[B32] Wahlund L. O. Barkhof F. Fazekas F. Bronge L. Augustin M. Sjögren M.. (2001). A new rating scale for age-related white matter changes applicable to MRI and CT. Stroke J. Cereb. Circ. 32, 1318–1322. 10.1161/01.STR.32.6.1318 11387493

[B33] Werring D. J. (2011). Cerebral Microbleeds: Pathophysiology to Clinical Practice, 1st ed. Cambridge, MA: Cambridge University Press.

[B34] Werring D. J. Gregoire S. M. Cipolotti L. (2010). Cerebral microbleeds and vascular cognitive impairment. J. Neurol. Sci. 299, 131–135. 10.1016/j.jns.2010.08.034 20850134

[B35] Zonneveld H. I. Goos J. D. C. Wattjes M. P. Prins N. D. Scheltens P. van der Flier W. M.. (2014). Prevalence of cortical superficial siderosis in a memory clinic population. Neurology 82, 698–704. 10.1212/WNL.0000000000000150 24477113

